# Midwifery Extraordinary

**Published:** 1882-12-20

**Authors:** J. Hendree

**Affiliations:** Alabama


### MIDWIFERY EXTRAORDINARY.

By J. HENDREE, M. D., OF ALA.

On the 15th of August last, a child was born in Selma, Ala. On the 17th a physician was sent for who found the infant in the following condition: It had a fracture of the right fibula with partial dislocation of the ankle outwards. Two fractures of the femur, one in lower third; the other above the middle. Fracture of left fibula with outward dislocation of ankle. Left femur broken at middle of shaft. Left forearm, both bones broken above the wrist. Left humerus, broken in middle of its length. On left side of trunk three dorsal ribs separated from the spine, on right all except the two first ribs likewise separated. There was a large contusion on right parietal about three inches in diameter. Since then the child has been fed from a bottle. Has had diarrhœa and dysentery, periodical colic requiring quinine. Has been afflicted with epidemic ophthalmia; had an attack of bronchitis needing special medication. October 16th, with slight deformity, the child was doing perfectly well and the case discharged.

The mother was too ill to be aware of the post natal accidents; the midwife, a white woman, is old, large, clumsy and totally blind. To obtain any information from her is impossible. When questioned, she goes into almost hysterical paroxysms of grief, wringing her hands and refusing to answer. The only supposition of the very capable physician who was called in, forty-eight hours after delivery, is that she let the infant slip from her hands to the floor, and, in grasping, fell across or upon it, crushing and producing the injuries described; which after all, are not so remarkable as the recovery of the child.

If desired, further particulars may be obtaiued from Dr. Jett. T. West, Selma, Ala.

